# Students’ motivational trajectories in vocational education: Effects of a self-regulated learning environment

**DOI:** 10.1016/j.heliyon.2024.e29526

**Published:** 2024-04-10

**Authors:** Tanja Held, Mathias Mejeh

**Affiliations:** a School of Education, Liverpool John Moores University, United Kingdom; b Zurich University of Teacher Education, Zurich, Switzerland; c Department of Research in School and Learning, Institute of Educational Science, University of Bern, Switzerland

## Abstract

Motivation is central for successful learning processes and lifelong learning. In the present study, the motivational development of vocational students in a learning environment promoting self-regulated learning (SRL) was examined in comparison to a control group with regular, teacher-centered instruction. The first aim was to examine the development of the dispositional and situational motivation of vocational students. The second aim was to gain a deeper understanding of the students' motivational experiences and the factors of the learning environment that promote and impede motivation. For this purpose, a mixed-methods design was applied. Through multilevel analysis, we investigated the development of dispositional motivation between the beginning and the end of the first year of vocational education (*N* = 159), as well as the development of situational motivation over the first 14 weeks using weekly motivation measures (*N* = 119). In addition, we interviewed 19 students from the SRL-promoting and regular school settings. The quantitative results revealed significant changes in dispositional and situational motivation over time. The qualitative results showed that the three basic psychological needs (relatedness, competence, and autonomy) were central determinants of the students’ motivation in both learning environments—albeit to varying extents. Overall, the SRL-promoting learning environment has positive effects on student motivation, but interindividual differences must be considered. Moreover, the results shed light on the coexistence of different motivation regulations within students and interindividual differences in the interpretation of the satisfaction of the three basic psychological needs.

## 1 Effects of a self-regulated learning environment on students’ motivational trajectories in vocational education

Motivation is a crucial prerequisite for successful learning processes and is indispensable for academic achievement, lifelong learning, and thus, active participation in society [1,2]. Despite the importance of motivation, several findings have revealed a decline in students' motivation in compulsory education and especially in lower secondary education [3, 4]. However, less is known about students' motivation development in vocational education^1^ (i.e., upper secondary education; [5]), with existing research in this context giving inconsistent results [5, 6]. Therefore, more longitudinal research is needed to gain insights into students’ motivational development in vocational education.

Given the importance of motivation for academic success, several interventions to foster motivation have been developed [7]. However, these interventions have mostly been produced in the context of compulsory or university education, while interventions for vocational schools are scarce [8]. Therefore, the first aim of the present study was to examine the development of vocational students' motivation in the first year of vocational school, and the second aim was to investigate whether a self-regulated learning (SRL) setting may have an impact on the development of students' motivation. To gain deeper insight into students’ motivational development, annual and weekly investigations of their motivation and qualitative data were used.

## 2 Fostering motivation through self-regulated learning settings

Given the evolving contexts, demands, and trends of the 21st century, educational institutions must empower students to acquire the adaptive skills and knowledge necessary for success in an dynamic world [9]. In this context, SRL emerges as a pivotal skill. Specifically, SRL is a self-directed process encompassing cognitive, affective, and behavioral dimensions, whereby students actively monitor and regulate their thoughts and behaviors to enhance their skills and knowledge [[10], [11], [12]]. In particular, Zimmerman's [13] social-cognitive model of SRL includes three cyclical phases: forethought, performance, and self-reflection. During the forethought phase, students assess the task, establish goals, and develop strategies to achieve them. During the performance phase, students actively engage in the task while continuously monitoring the progress. Finally, in the self-reflection phase, students evaluate their learning outcomes and adjust their future learning activities accordingly [13]. These three phases are linked by feedback loops, thus allowing students to adapt to changes in the environment and learning outcomes (for a more detailed description, see [13]).

SRL competencies are crucial for transitioning from educational environments to work settings and for achieving and retaining employment, especially in the context of vocational education [[14], [15], [16]]. For example, Kirschner and Stoyanov [14] stated that metacognition and reflection are key skills for future jobs and lifelong learning and that the educational curricula should be revised accordingly. This suggestion is in line with previous calls for education reforms to prepare future generations for navigating in a technologically advanced and competitive world [17]. Therefore, the promotion of SRL has an important role in different areas of the educational system, where students must be prepared for the demands of work and lifelong learning [18,19].

The essential role of motivation in SRL has been elaborated on both theoretically and empirically by Paul Pintrich [[20], [21], [22]]. Indeed, since being identified as a key component of successful SRL, motivation has been the subject of numerous studies, which have consistently revealed a positive relationship between SRL and motivation (for an overview see [23]). Motivation as an essential component of SRL also plays a crucial role for learning success in VET [[24], [25], [26]] and retention of employment [27,28]. Specifically, motivational processes play a fundamental role in education as they affect cognitive processes, emotions, and learning outcomes of vocational students [[29], [30], [31]]. Furthermore, Rozendaal [32] demonstrated that affective variables in SRL are influenced by the quality of the learning environment. For example, direct interventions can increase vocational students’ motivation while reducing anxiety, leading to deeper information processing [30]. Finally, an increased sense of autonomy in vocational students also positively impacts their learning motivation and their ability for self-regulation in learning [33,34].

The importance of autonomy in learning is also reflected by Deci and Ryan's (2002) self-determination theory (SDT), which assumes that the fulfillment of three basic psychological needs (autonomy, competence, relatedness) is critical to perceiving a behavior as self-determined [[35], [36], [37], [38]]. Specifically, competence refers to effectiveness and mastery, autonomy to volition and willingness, and relatedness to interpersonal relationships [39]. When basic psychological needs are better fulfilled, individuals experience greater self-determined motivation, as reflected in the continuum perspective of motivation, which ranges from intrinsic motivation to different forms of extrinsic motivation [37]. On this continuum, the types of motivational regulation (see Table 1) differ in their degree of self-determination and internalization into the self [40]. Intrinsic and extrinsic motivation involve the intention to engage in a certain way, whereas amotivation reflects the absence of such intention and lies outside the continuum [37].

**Table 1:** Summary of motivation regulation according to SDT [37,41].

Behavior	Motivation	Regulation style	Description
Self-determined	Intrinsic motivation	Intrinsic regulation	The state of demonstrating a behavior because of the activity itself and inherent satisfaction, is regarded as the prototype of self-determined behavior, as it reflects the individual's interests.
	Extrinsic motivation	Identified regulation	Behavior is considered important and integrated into the self and fulfills an instrumental purpose (e.g., learning to be able to do a certain education later).
Non-self-determined		Introjected regulation	The behavior does not require any external impulses, but it is only slightly internalized and is primarily based on feelings like shame, guilt, or pride.
		External regulation	The behavior is performed based on external contingency (e.g., reward or punishment).
	Amotivation	Non-regulation	The lack of intention to act.

Overall, both SRL and SDT emphasize that social and contextual factors influence individuals and their SRL and learning motivation. Perry, Mazabel [42] synthesized the classroom practices that support SRL and learning motivation based on SDT, with the four identified macro categories (see Table 2) reflecting the classroom characteristics that tend to be associated with SRL and autonomous motivation [42]. It can be assumed that instructional practices based on these classroom characteristics should simultaneously promote SRL and autonomous motivation (for further information on synergies between SRL and SDT see [42]).

**Table 2:** Classroom characteristics for enhancing self-regulated and self-determined learning (adapted from [[42], [43]]).

Macro Categories	Micro Categories	Classroom Characteristics
**Providing Structure**	Tasks/Activities	Tasks and activities are designed to meet various instructional objectives and are relevant and authentic.
	Expectations/Instructions	Instructions are discussed and/or collaboratively developed.
	Familiar Routines and Participation Structures	Consistent, established norms and guidelines for engaging in activities.
	Accommodations for Individual Differences	Activities and assessment approaches are adaptable to accommodate different interests and abilities.
	Visual Prompts	Visual prompts induce students to learn.
**Giving Students Influence**	Involvement in Decision Making Choices	Students actively participate in decision-making regarding their learning.
	Control over Challenge	Students are responsible for adjusting the difficulty level of activities.
	Self-Assessment	Students can evaluate the quality of their effort and decide on their subsequent actions.
**Supporting, Scaffolding, Co-Regulation**	Modeling/Demonstrating	Explaining the steps required to accomplish the task.
	Questioning	Employing questions to direct learning and encourage students to find solutions.
	Feedback	Providing students with feedback targeting the learning process.
	Metacognitive Language	Promoting the use of metacognitive language.
	Motivational Messages	Attributing success to effort and applying strategies, highlighting progress and development.
**Creating a Community of Learners**	Co-constructing Knowledge	In the process of knowledge construction, teachers collaborate with students as learning partners.
	Positive/Non-Threatening Communication	Teachers and students communicate with each other respectfully.
	Supporting/Celebrating One Another's Learning	Students are motivated to engage in collaborative work.

## 3 Motivation from a time perspective

Dispositional motivation can be defined as a construct that influences an individual's general or habitual responses to specific events. Therefore, this type of motivation can be considered as a trait or disposition and “a source of energy and direction for an action in relation to a general class of situations or across a range of like contexts” [44]. Conversely, situational motivation is an ongoing process that results from the current person-situation interaction [45,46]. Consequently, existing research on motivation must be differentiated and interpreted based on the type of motivation measured.

To investigate motivation development, existing research often collected data over large time intervals (e.g., every academic year: [6]) and, thus, focused on changes in dispositional motivation (i.e., coarse-grained measurement; [[44], [46], [47], [48], [49]]). In contrast, different fine-grained measures of motivation [[49], [50], [51], [52]] investigate motivation as a dynamic process (situational motivation), revealing that motivation may increase, decrease, or fluctuate across time. Most intraindividual fluctuation in motivation has been found between days [53,54], whereas less variability has been found over weeks and months [51]. Additionally, individual factors (e.g., competence beliefs), task and contextual aspects are relevant predictors for intraindividual motivation fluctuations [[50], [51], [52], [53],^55^,^56^]. This process perspective for understanding motivation indicates that motivation is a dynamic phenomenon and that students experience variations in motivation based on situational factors [52]. Furthermore, situational motivation is assumed to underlie dispositional motivation, implying, for example, that repeated experiences of situational motivation contribute to dispositional motivation (see also the hierarchical model of intrinsic and extrinsic motivation [57]).

Regarding dispositional motivation, existing research has revealed a general decline in students’ motivation across school years (e.g., [[3], [4], [35], [58], [59]]). However, for vocational schools, research has provided different results. Specifically, Gurtner, Gulfi [6] found a continuous motivational decrease over 3 years, whereas van der Veen and Peetsma [5] found no significant effect on motivation over the first year of vocational education. A prominent explanation for the decrease in dispositional motivation observed in adolescence (e.g., [[3], [35]]) is provided by the stage-environment fit theory (SEFT; [60]), which is related to the SDT. In particular, SEFT assumes that the fit between the needs of adolescents and the school context deteriorates over time, and the increasing mismatch leads to a decrease in motivation [61].

Similarly, research on situational motivation has also revealed that fulfilling the basic psychological needs of students affects their situational motivation [36,54,56]. However, further research on the development of situational motivation is needed––especially in vocational education––because little research has examined the short-term dynamics of motivation [[62], [63], [64]].

## 4 Aims of the present study

Prior research on student motivation in vocational education has generally analyzed motivation as a disposition and focused on pre- and post-measurements to provide information on the development of motivation (coarse-grained measurement; [[5], [6]]). However, empirical studies focusing on fine-grained measures of motivation are lacking in vocational education research. Given that motivational processes develop over time and may exhibit regular trends or patterns, small-scale analyses are also essential for explaining and predicting student motivation. By applying such a process-oriented perspective, a more detailed understanding of the dynamic characteristic of motivation within students can be obtained [52,56,65], thus supporting the understanding of how dispositional changes occur [64]. Therefore, the present study aims to combine fine- and coarse-grained measurements (weekly vs. annual measurements) of motivation to examine motivation changes in vocational education.

The present study quantitatively examines the regulation of dispositional and situational motivation and amotivation over time and analyzes whether an SRL-promoting learning environment affects the motivation development of vocational students.

- Regarding vocational students' dispositional (H1a) and situational (H2a) motivation development, we assume that intrinsic and identified regulation may increase in the SRL setting group.
- Based on the stronger internalization according to the SDT (e.g., [66]), we hypothesize regarding introjected, external regulation, and amotivation a decrease in dispositional (H1b) and situational motivation development (H2b) in the SRL setting group.
- Based on the inconsistency of prior findings in vocational education [5,6], we hypothesize no change in the students' dispositional (H1c) and situational motivation development (H2c) in the control group.

Furthermore, to gain a deeper understanding of students' motivational experiences in the two learning settings (SRL-promoting vs. regular vocational school setting), we analyze qualitative interview data. These interviews will allow the exploration of how students describe their motivational experiences in the two settings in vocational education and which factors of the vocational school setting promote or impede motivation. Finally, we aim to evaluate the experiences and factors mentioned in the interviews to better understand students’ motivational development in vocational education.

By combining qualitative and quantitative strands of research, the present study applies a mixed-methods sequential research design [67] and acknowledges the necessity for qualitative research in the field of motivation [68]. Moreover, combining the two methodological research approaches allows for the development of broader and deeper insights [69]. Indeed, to capture the complexity of motivational development, it is necessary to explore both the situation and the structure of action.

To summarize, the present study aims to contribute to the following.

- gaining a deeper understanding of motivation in vocational education, which is underrepresented in existing research, by combining quantitative and qualitative data;
- investigating how an SRL-promoting learning environment affects vocational students' motivation;
- highlighting the difference between coarse- and fine-grained measures of motivation and how they can complement each other.

## 5 Method

### 5.1 Participants and procedure

The current study is part of the project “Selbstgesteuertes Lernen in der Berufsbildung” (self-regulated learning in vocational education) and uses a quasi-experimental design with an SRL setting group (three classes, *n* = 68; 42.8 %) and a control group (four classes, *n* = 91; 57.2 %). Students involved in the study voluntarily opted to join the SRL setting classes. In Switzerland, vocational school students attend classes on 2 days per week. During the other 3 days, the students work at apprenticeship companies to acquire the skills to become commercial employees. Our study and research questions solely addressed learning in school rather than in the apprenticeship companies.

**Fig. 1:**
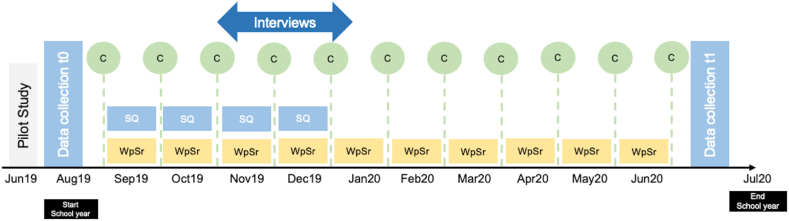
Study Design *Note.* DQ = dispositional questionnaire; C = coaching session; SQ = situational questionnaire; WpSr = weekly planning and self-reflection form, t0 = first dispositional measurement point; t1 = second dispositional measurement point.

The quantitative sample comprised 159 students (76 male, 83 female; mean age = 16.65 years, *SD* = 2.23). Two main self-reported questionnaires were administered to the students at the start (August) and the end of the school year (June). In addition, the students could participate in small weekly questionnaires (see Fig. 1). 46 students in the SRL setting and 73 students in the regular school setting participated in the weekly situational questionnaire over a 14-week period (one semester) using an application designed for this study. The application sent a push notification to students’ smartphones once a week to inform them of a new questionnaire. A semi-randomized time interval was used for data collection to eliminate any possible bias due to a time or day [70]. The weekly questionnaire took students approximately 1–2 min to complete. Due to the school closure during the COVID-19 pandemic, there was no weekly survey in the second semester.

To gain deeper insights during the quasi-experimental phase, we conducted semi-structured interviews to substantiate our quantitative findings. Therefore, qualitative data from 19 students (*n*_SRL setting_ _class_ = 12; *n*_control class_ = 7; male = 8; female = 11) were collected between November and January. Using an extreme case sampling method, students were selected for interviews based on their measured motivation at the beginning of the school year, considering both high and low motivated students [71].

The study received full ethical approval by the Ethics Committee of the Faculty of Human Science of the University of Bern (reference: 2019-07-00001). Quantitative and qualitative data collection were piloted before the main study took place. Permission was obtained from parents and students. All student-provided data were anonymized.

### 5.2 SRL-promoting setting

According to the findings of Perry, Mazabel [42], the four identified macro categories (see Table 1) simultaneously promote SRL and autonomous motivation. Therefore, we developed our SRL setting based on this framework model. The elements of the SRL setting and their links to the macro and micro categories of classroom practices are described in more detail below (see also [72]).

Students in the SRL setting received no regular lessons during the two school days. Instead, they attended four to five 20-min inputs each day (short lectures by teachers). These sessions covered the technical content of subjects such as German, Information, English, Business and Society, French, and Communication, and Administration. In the remaining time, students could work independently on individual tasks. Two teachers were available throughout the school day to answer the students’ questions, and on the second school day (from 09:00–10:00am) an exam was scheduled each week. All tasks, exam dates, and optional self-tests were listed in the so-called “learning job,” which covered 4 weeks at a time. Using these learning jobs, students made individual plans for each week, autonomously determining the allocation of time for each subject or task both at school and at home. Moreover, a teacher accompanied each student. During these coaching sessions, which occurred at least once a month, coaches and students reflected on their planning and implementation of the learning jobs and weekly plans.

The learning jobs and 20-min inputs primarily addressed the macro category “Providing Structure” by providing authentic and meaningful tasks (Tasks), explanatory rationales (Instruction), practicable routines (Routines and Participation Structures), opportunities for all students to participate (Accommodations for Individual Differences), and visual cues (Visual Prompts). Additionally, the students’ weekly plans provided opportunities for the students to influence the learning process (Giving Students Influence) by allowing them to engage in the learning process and make choices (Involvement in Decision-Making Choices), control the challenge through varying levels of difficulty (Control over Challenge), and complete optional self-assessments (Self-Assessment). The macro category “Supporting, Scaffolding, Co-Regulation” was addressed by the coaching sessions, with the teachers serving as supporters of learning. Finally, the macro category “Creating a Community of Learners” was addressed by giving the opportunity to students to collaborate with peers for each task within the learning jobs (see also [72]).

In sum, the SRL setting covered all categories identified by Perry, Mazabel [42], and thus, could be defined as a learning setting in which both SRL and autonomous motivation were promoted. In contrast, students in the control classes attended regular lessons (90 min) dedicated to a specific subject (e.g., German or Business and Society) on the two days of school. At the end of each lesson, they moved to a different classrooms and a different teacher. Teachers were primarily responsible for the instructional design, which differed across subjects. The students in the control classes also had to complete homework between lessons in each subject. In addition, exams were scheduled (approximately every four to six weeks) with no coordination of exam dates between subjects. This resulted in students in the control group having two or three exams in the same week. None of the instructional components offered to the students in the SRL setting (e.g., coaching or weekly plans) were implemented in the regular classes.

### 5.3 Measures

#### 5.3.1 Dispositional questionnaire

The students' dispositional motivation was assessed using the adapted version of the German Self-Regulation Questionnaire [73] developed by Ryan and Connell ([74] see Table 3). Ratings for all items were provided on a five-point Likert scale (1 = *strongly disagree*; 5 = *strongly agree*). Intrinsic regulation was assessed with five items, identified and introjected regulation with four items, and external regulation with six items. Furthermore, students' amotivation was measured on a five-point Likert scale (1 = *strongly disagree*; 5 = *strongly agree*) that comprises three items ([73]; for full questionnaire, see Appendix A). The Cronbach's alpha values of all scales were in line with the validated scales (see [[73], [75]]).^2^

**Table 3:** Scales and Items. Each Example Item Represents the Item Used in each Weekly Situational Questionnaire.

Scale and Example Item (Number of Items in Dispositional Questionnaires)	α_t0/t1_
Motivational regulation (Müller et al., 2007)	
Intrinsic regulation (5 items) ‘I work and learn in vocational education because I want to learn new things.’	81/.85
Identified regulation (4 items) ‘I work and learn in vocational education because the things I learn here will be useful for my later job.’	0.77/.84
Introjected regulation (4 items) ‘I work and learn in vocational education because otherwise I would have a guilty conscience.’	0.63/.72
External regulation (6 items) ‘I work and learn in vocational education because otherwise I would get into trouble at my training company.’	0.64/.81
Amotivation (Thomas & Müller, 2011; 3 items)	
‘If the teacher doesn't notice it, I occupy myself with other things.’	0.61/.84

*Note:* t0 = first dispositional measurement point (August); t1 = second dispositional measurement point (June).

The concurrent validity between the two groups was assessed for each scale. The item correlation matrix was similar in both groups, and the confirmatory factor analysis revealed similar results in both groups. In addition, measurement invariance between the groups was tested for the dispositional variables [76]. Changes in model fit were assessed using ΔCFI (≤0.01), ΔRMSEA (≤0.015), ΔSRMR (≤0.30 for metric and ≤0.015 for scalar invariance), and *p*-values of Δχ^2^ (see [77]). Measurement invariance revealed scalar invariance between the groups for all the variables.

#### 5.3.2 Weekly situational questionnaire

For the weekly situational questionnaire, single items from the dispositional questionnaire were used. When long scales are not feasible, single-item measures can be used as they have sufficient psychometric properties [78]. We used items from the dispositional questionnaire as single items (see Table 2). We carefully selected items that most accurately reflected each scale, guided by criteria such as convergent validity and face validity (see [[79], [80], [81]]). For the situational questionnaire, all items were measured on a four-point Likert scale (1 = *strongly disagree*; 4 = *strongly agree*) to avoid changes in the response scales in the weekly questionnaire, as these consisted of different questions (e.g., questions regarding learning strategies).

#### 5.3.3 Interviews

To explore students' motivational experiences, this study also employed a qualitative approach. Semi-structured face-to-face interviews by members of the research team were conducted. The interviews were recorded and, according to predefined transcription rules, transcribed verbatim. The interviews took place in the school and lasted between 23 and 46 min. The interviews were carried out in an informal, conversational style, aimed at fostering open discussion of the students’ experiences and opinions.

The interview guide included questions about the students' applications of (meta-)cognitive strategies, as well as their perceived motivational and emotional states (for central questions on motivation, see Appendix A; Tables S1 and S2). Moreover, the students in the SRL setting were asked to evaluate the SRL-promoting setting in terms of its potential and challenges for their individual learning processes, whereas students in the control group were asked to reflect on the challenges they experienced in the regular learning setting and outline ideas for improvements that could be made. For this analysis, questions about the students’ motivational experiences and factors promoting or impeding motivation were of particular interest. Follow-up questions were used to encourage elaboration on the responses, and students were encouraged to substantiate their answers with examples. All personally identifiable information was removed, and each transcript was anonymized by assigning a code.

### 5.4 Data analyses

#### 5.4.1 Quantitative analyses

Firstly, we wanted to investigate the development of dispositional motivation between students in the SRL setting and control classes. The dispositional data corresponded to a nested data structure, with measures nested within individuals and within classes. Therefore, we conducted linear mixed-effect models using the dispositional data collected at the beginning and end of the school year.

Secondly, to investigate the development of situational motivation, we analyzed weekly situational data comprising 14 measurement points. The situational data also represented a nested data structure. The nested data structure was included if the ICC was >0.10 in order to avoid unnecessarily complex models.

To explore the development of the variables (dispositional and situational), we first established a baseline model, comprising only the intercept. Subsequently, we included varying intercepts across students and time as predictor. In the third model, the intercepts and the effect of time were allowed to vary across students (random intercept and random slopes). Finally, if there was a significant development over time, an overall model was fitted to examine whether students in the SRL setting differed from those in the control group (interaction model; model 4). We used the *nlme* package (version 3.1.152; [82]) in R [83] to conduct the analyses.

With frequent measurements, missing data is a common challenge. In longitudinal research, the use of contemporary statistical procedures, such as maximum likelihood estimation, is recommended for handling missing data [[84], [85], [86]]. Missing data occurred because participation in the study was voluntary and because of absences (e.g., illness) during the survey period. Missing data in the dispositional questionnaire (t0 and t1) and in the situational questionnaire were estimated using ML estimation. The percentage of missing values varied between 17 % and 40 % for the dispositional questionnaire and between 34.45 % and 82.35 % for the situational questionnaire. On average, 65.12 % of the students participated in the weekly measurements.

#### 5.4.2 Qualitative content analysis

We used qualitative content analysis [87] to identify the perceived motivational experiences and factors promoting or impeding motivation based on SDT. For the coding process, we used the software MAXQDA [88]. The two authors double-coded 2 of the 19 interviews, and the intercoder reliability was sufficient (corrected Cohen's kappa = 0.66; [89]). For the coding schema, we built deductive categories based on SDT [41], with seven main categories. The category system can be found in Appendix A (Table S3).

## 6 Results

Table 4 displays the descriptive statistics and correlations of the dispositional variables. As expected, all variables correlated both within and across measurement points, with significant correlations aligning with the expected direction. Variables of the motivation regulation continuum (intrinsic to external regulation) were significantly correlated within a time point (e.g., intrinsic and identified regulation at the first measurement point). In addition, the variables at the first measurement point were correlated with themselves at the later time point. At the baseline measurement, the two groups demonstrated no significant differences in terms of any of the motivational variables (intrinsic regulation: *t*(130) = 0.78, *p* = .44; identified regulation: *t*(129) = -0.20, *p* = .84; introjected regulation: *t*(130) = -0.01, *p* = .99; extrinsic regulation: *t*(128) = -1.16, *p* = .13; amotivation: *t*(130) = -0.53, *p* = .60), age (*t*(132) = -0.20, *p* = .85), or gender (*t*(130) = -0.80, *p* = .43).

**Table 4:** Mean, standard deviations, and correlation of the dispositional questionnaire.

	Variables	*M*	*SD*	1	2	3	4	5	6	7	8	9	10
1	Intrinsic regulation t0	3.15	0.74	1	0.28**	0.24**	−0.02	0.24**	0.18	−0.06	0.03	−0.27**	−0.05
2	Intrinsic regulation t1	3.10	0.74		1	0.12	0.24**	0.05	0.25**	−0.04	0.12	−0.11	−0.06
3	Identified regulation t0	4.33	0.61			1	0.20*	0.19*	0.15	0.27**	0.10	−0.11	−0.09
4	Identified regulation t1	4.11	0.82				1	0.06	0.22**	0.00	0.27**	−0.02	−0.16
5	Introjected regulation t0	2.61	0.88					1	0.22*	0.27**	0.07	−0.06	−0.10
6	Introjected regulation t1	2.80	0.95						1	0.06	0.36**	−0.05	0.07
7	External regulation t0	3.44	0.63							1	0.23*	0.13	0.06
8	External regulation t1	3.38	0.81								1	0.02	0.13
9	Amotivation t0	1.67	0.60									1	0.20*
10	Amotivation t1	2.01	0.96										1

*Note.* *p < .05, **p < .01. Min = 1; Max = 5; t0 = first dispositional measurement point (August); t1 = second dispositional measurement point (June).

### 6.1 Dispositional motivation development

Separate linear mixed models were conducted to examine differences in students’ motivational development over the first year of vocational education in the SRL setting and control classes (H1a, H1b, and H1c). If there was a significant development over time, an overall model was fitted to examine whether students in the SRL setting differed from those in the control group. Appendix B presents the model parameters and the goodness-of-fit indices of all motivational variables for both groups. The results presented in Table 5 correspond to the best-fitting model (indicated by bold markings in Tables S1–S7 in Appendix B).

**Table 5:** Summary of significant results of dispositional motivation development.

	Identified regulation		Amotivation	
	SRL setting group	Control group	SRL setting group	Control group
	*B (SE)*	*B (SE)*	*B (SE)*	*B (SE)*
Fixed effects				
Intercepts	4.31 (0.08)***	4.34 (0.08)***	1.64 (0.07)***	1.70 (0.08)***
Time^1^	−0.07 (0.09)	−0.33 (0.13)*	0.37 (0.16)*	0.40 (0.12)**

Note. *p < 0.05; **p < 0.01; ***p ≤ 0.001. ^1^(0 = start of vocational school [August], 1 = end of first year of vocational school [June]).

In the control group, a significant effect of time was found in identified regulation (*b* = −0.33, *t*(35)= −2.48, *p* = 0.018). Specifically, identified regulation decreased significantly in the control group over the first year of vocational education, whereas it remained stable in the SRL setting group (*b* = −0.07, *t*(33) = −0.74, *p* = 0.46). The overall model revealed no significant interaction between setting and time. In terms of amotivation, separate models showed a effects of time in the SRL setting group (*b* = 0.37, *t*(33) = 2.37, *p* = 0.024) and in the control group (*b* = 0.40, *t(35)* = 3.20, *p* = 0.003). In particular, amotivation increased significantly in the SRL setting and control groups over the first year of vocational education. The overall model revealed no significant interaction between the setting and time. We found no significant differences in amotivation between the SRL setting and the control group over time. There was no time effect for any of the other motivation variables, neither in the SRL setting nor in the control group.

Overall, regarding the development of motivation in the SRL setting group, hypotheses H1a (increase in intrinsic and identified regulation) and H1b (decrease in introjected, external regulation, and amotivation) must be rejected. No changes were expected in the control group, and thus, hypothesis H1c must be partially rejected, as a significant decrease in identified regulation and a significant increase in amotivation was found.

### 6.2 Situational motivation development

Separate linear mixed models were conducted to examine the situational development of motivation in the SRL setting and control group (H2a, H2b, and H2c). If there was a significant development over time, an overall model was fitted to examine whether students in the SRL setting differed from those in the control group. Appendix C presents the model parameters and the goodness-of-fit indices for both groups. Table 6 presents the best-fitting model (indicated by bold markings in Tables S1–S7 in Appendix C).

**Table 6:** Summary of significant results of situational motivation development.

Effect	Identified regulation			Introjected regulation		
	SRL setting group	Control group	Overall group	SRL setting group	Control group	Overall group
	*B (SE)*	*B (SE)*	*B (SE)*	*B (SE)*	*B (SE)*	*B (SE)*
Fixed effects						
Intercepts	3.13 (0.07)***	3.14(0.10)***	3.10 (0.19)***	2.60 (0.21)***	2.77 (0.12)***	2.29 (0.30)***
Time (weeks)	0.01 (0.01)	−0.02 (0.01)*	0.01 (0.01)	0.03 (0.01)**	0.01 (0.01)	0.03 (0.01)*
SRL setting			0.02 (0.12)			0.24 (0.19)
Time x SRL setting			−0.03(0.02)*			−0.02 (0.02)

*Note.* *p < 0.05; **p < 0.01; ***p ≤ 0.001.

**Fig. 2:**
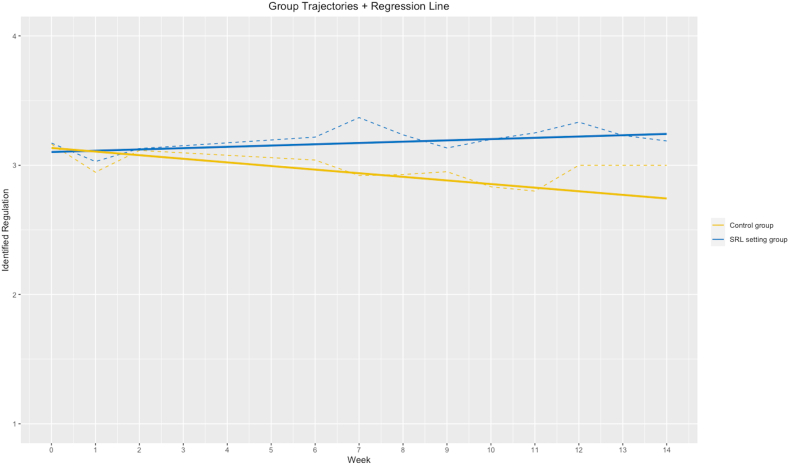
Situational Development: Identified Regulation *Note.* Dashed line = aggregated values per measurement point; solid line = regression line; blue line = SRL setting group; yellow line = control group.

Results showed that identified regulation decreased in the control group (*b* = −0.02, *t*(201) = −2.34, *p* = 0.02). In the SRL setting group, no significant change over time was observed (*b* = 0.01, *t*(208) = 0.95, *p* = 0.34). In the overall group, the findings showed a significant interaction effect between time and setting in identified regulation (*b* = −0.03, *t*(409) = −2.30, *p* = 0.02). This indicates a significant difference in slopes between the two groups over time (see Fig. 2).

**Fig. 3:**
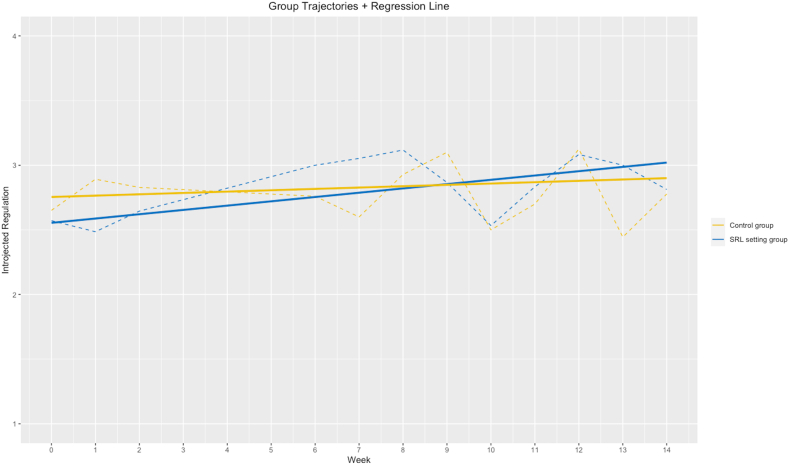
Situational Development: Introjected Regulation *Note.* Dashed line = aggregated values per measurement point; solid line = regression line; blue line = SRL setting group; yellow line = control group.

Moreover, we found a significant time effect in introjected regulation in the SRL setting group (*b* = 0.03, *t*(207) = 2.91, *p* = 0.004). In the control group, no significant effect was observed (*b* = 0.01, *t*(201) = 0.79, *p* = 0.43). This effect suggests that the introjected regulation of the students in the SRL setting significantly increased over time (see Fig. 3). The overall model revealed no significant interaction between the setting and time, but there was a main effect of time (*b* = 0.03, *t*(408) = 2.24, *p* = 0.03). In both groups, no significant main effects of time were found in intrinsic regulation, external regulation, or amotivation (see Appendix C).

Overall, regarding the development of motivation in the SRL setting group, hypotheses H2a (increase in intrinsic and identified regulation) and H2b (decrease in introjected regulation, external regulation, and amotivation) must be rejected. No changes were expected in the control group, and thus, hypothesis H2c must be partially rejected, as a significant decrease in identified regulation was found.

### 6.3 Qualitative data

In total, eleven codes were assigned to seven main categories. Firstly, the results are presented and interpreted along the five motivational dimensions: intrinsic regulation, identified regulation, introjected regulation, external regulation, and amotivation. Secondly, the analysis and classification of the factors promoting and impeding motivation are provided. To increase readability, the student quotes have been cleaned of repetitions, pauses, and filler words.

#### 6.3.1 Motivational experiences

##### 6.3.1.1 Intrinsic regulation

The statements made by the students in the SRL setting regarding intrinsic regulation highlighted positive learning emotions, interest, and an awareness of one's learning development. In particular, enjoyment of the job and school seem to be important drivers of students' learning behavior:

“I enjoy every subject – I can honestly say that I'm interested in everything.” (S_5_T, pos. 35)^3^

Similar statements were also found in the interviews of the control group. Specifically, the students expressed interest in learning something new but also emphasized that there was no pressure to attend vocational school and that they pursued vocational education for themselves.

“Yes, because it is actually voluntary that I have chosen this apprenticeship. That's why. I think that's enough motivation.” (S_13_C, pos. 52)

##### 6.3.1.2 Identified regulation

In terms of identified regulation, students in the SRL setting primarily emphasized the instrumental purpose of instruction. In particular, learning was linked to a superordinate goal, but these goals differed among the students. For example, some expressed that it is important to pass vocational education for active participation in society, as well as to graduate with high grades in order to pursue higher education later:

“Also, of course, that I want to finish the apprenticeship with good grades. So, for me that's very important because I don't know if I might want to go to university later or something.” (S_8_T, pos. 42)

Moreover, the students also attributed inherent value to the SRL setting. Learning engagement can be constructed as an action that is directed toward not having to leave the SRL class.

For identified regulation, no major differences were observed in the control group. Indeed, this group also focused on the overarching goal of successfully completing the apprenticeship, and the future value of learning was reflected by the fact that the students perceived the learning content as being relevant beyond vocational education:

“… learning things and just being able to use them after the apprenticeship.” (S_16_C, pos. 44)

##### 6.3.1.3 Introjected regulation

In the SRL setting, introjected regulation appeared primarily in the form of a guilty conscience and stress. The students reported negative emotions occurring especially when they postponed their learning behavior despite knowing the importance of learning and preparing for upcoming exams:

“So, I get stressed about it. If I plan that I want to do it on Thursday or Friday and then I say, ‘No, I'll do it on Saturday, Sunday, Monday’ and then it's still not done. And then I am stressed because I know I should do it so that I can prepare well for the next test.” (S_7_T, pos. 62)

However, these students also showed introjected regulation by the fact that they had an unconditional intention to make a good impression on their parents, the apprenticeship company, and their teachers:

“Well, I want to make a good impression on my apprenticeship company. I want to make a good impression at home and with the teachers.” (S_8_T, pos. 34)

Similar patterns of justification emerged in the interviews with the students in the control setting:

“I always tell myself that I do it for myself and not for others. And that I actually have the problem afterwards if I don't learn and I can't blame it on someone else.” (S_18_C, pos. 36)

##### 6.3.1.4 External regulation

In terms of external regulation, students in the SRL setting highlighted two external aspects that influence their learning behavior. Firstly, the constant evaluation from teachers, coaches, parents, and the apprenticeship company was reported to be crucial for the students. Both positive (e.g., praise) and negative (e.g., criticism) feedback from one of these actors was mentioned as motivating:

“The feedback from the coach is enormously motivating, I have to admit, because my coach gives good feedback and she also gives feedback on the learning job itself, what I did well, what I didn't do well, what I have to pay attention to, and that simply motivates me.” (S_5_T, pos. 103)

Secondly, summative feedback (i.e., grades) was emphasized by the students. The students gave high relevance to grades, expressing that they wanted to achieve either good or sufficient grades in a subject based on their past achievements. The students associated negative emotions with poor grades, whereas good grades elicited positive emotions and represented a form of positive reinforcement. In the control group, formative and summative feedback were also relevant. In addition, some apprenticeship companies (in the SRL setting and control group) rewarded good grades at the end of each semester with money (bonuses):

“I know that if I work hard enough for school, it will show in the grades later, and that motivates me. Because I would like to get good grades. That makes me happy, it makes the company happy, it makes my family happy, and in our apprenticeship company, we get bonuses for good grades and that's also a bit of a motivation.” (S_13_C, pos. 154)

##### 6.3.1.5 Amotivation

In both groups, some students mentioned situations in which they had no intention to learn, including stressful events in and outside of school, negative emotions, and bad moods related to learning:

“When I'm in a bad mood, I can honestly say that learning is very difficult for me. And especially listening to the inputs. I just don't feel like it. I just want to go home. And then it's so hard for me to pay attention and do my stuff.” (S_4_T, pos. 352)

“For example, when I get home and something bad or stupid happens on the way, I think, ‘Oh, I'm not going to study now, no way.’ That can have a very strong impact on me and my motivation.” (S_19_C, pos. 180)

Moreover, the students were typically unable to name a strategy for overcoming this temporary amotivation. Indeed, the students often reported letting themselves be overwhelmed by their lack of intention to act until their unpleasant feelings dissipated and they regained their motivation.

#### 6.3.2 Promoting and impeding factors

##### 6.3.2.1 Autonomy

Students in the SRL setting described in detail that they perceived the SRL setting to be beneficial in terms of autonomy. In particular, they emphasized that setting their own goals gave them responsibility, the possibility of planning, and a sense of individualization. The students’ explicit references to the regular classroom underscored the advantages of the SRL setting in terms of the individualization of the learning pace and environment (e.g., learning with music). Students appreciated and benefited from the freedom and flexibility offered by the SRL setting and also recognized the importance of successful self-regulation for their professional lives.

However, students also cited the great autonomy of the setting as an impeding factor to their motivation. Indeed, they recognized that self-regulation competencies are crucial for successful development but also that this freedom can be abused. In this respect, students in the SRL setting seemed particularly sensitized and reflected on their self-regulation competencies with a consideration of the advantages and disadvantages of autonomy:

“I see that as an advantage. But I think that's why many students attend the normal setting because they don't have the prerequisites. For example, you must be independent from the beginning. […] you must make your plan, and no one tells you that you must do it now. […] You must want to learn, of course, and you cannot just sit down a little tired and simply let the lesson wash over you.” (S_7_T, pos. 180)

Students in the control group also identified the increased personal responsibility compared to compulsory school as a motivation-promoting factor. However, less focus was directed to the lessons and the time spent in class and more to self-study time at home:

“You just learn a little bit more how to deal with work and school and how to combine the two […].” (S_16_C, pos. 254)

Regarding the misfit of autonomy, some students in the control group indicated that they would like to have more autonomy:

“When it comes to test preparation, everyone can work on what they want. But compared to the SRL setting, we are less autonomous because it is only one lesson a week.” (S_17_C, pos. 37)

##### 6.3.2.2 Competence

Both groups indicated that the fit of the task difficulty to individuals’ competency was a motivating factor. The satisfaction of the need for competence serves as a positive reinforcer for learning behavior, which can also have an impact across different subjects:

“Because I have understood the task in economy and society—because I have done it myself, I have more joy to work on the other subjects.” (S_3_T, pos. 45)

In contrast, tasks that are too difficult cause negative emotions, which can lead to avoidance behavior and denial. In relation to these situations, students in both groups wanted more individualization. However, single non-fitting events (e.g., illustrated by a bad grade) could also serve as a motivator by encouraging the students to learn from the mistakes and to want to do better next time. Individual interpretation seems to be crucial in determining whether the misfit between task difficulty and competence negatively or positively affects their motivation:

“If, for example, the English grade is not good or if I get feedback that I didn't do something well, then it motivates me. I know that some people don't get motivated when you tell them what they didn't do well. […] For me, it is the other way round. It motivates me to be better so that next time I can show him that I can do better.” (S_15_C, pos. 149)

##### 6.3.2.3 Social relatedness

In terms of social relatedness, the students in the SRL setting particularly focused on the class climate as well as the support they received from their peers:

“Mostly I learn in small groups, i.e., with colleagues.” (S_12_T, pos. 4)

The students reported having many opportunities to cooperate and fulfill their personal need for social relatedness in the SRL learning setting. This learning environment seems to lead to greater class cohesion and a positive working atmosphere, which is an important component of successful group-regulated learning. The learning space (e.g., the possibility to move around freely) was also explicitly rated positively, as it allowed the students to exchange ideas and support each other:

“The arrangement of the room so that everyone can go to everyone a bit – not in rows where you have your fixed place, and I think that also makes a big difference. Because we walk around and help each other. If you only have your desk neighbor on the left, desk neighbor on the right, you will probably only ask them. So that's why, yeah, I think the interior plays a role too.” (S_10_T, pos. 103)

In this context, students also felt well-supported by the teachers and maintained positive relationships with them. The students felt “noticed” by the teachers and were supported in their learning processes. In addition, the relationship with the coaches was viewed positively, as they played an important role in the students' stress regulation. The coaching was perceived as useful when the students could participate in the structure and content of the coaching and were allowed to communicate their expectations and needs, and it seemed to be particularly helpful when solutions could be developed together. Finally, actors outside the classroom played an important role in the students’ learning. For example, students were sometimes supported in their exam preparation by family members, friends, or people in the apprenticeship company.

Support from teachers, peers, families, and the apprenticeship company also played a key role in the learning of the students in the control group. Overall, class cohesion was not emphasized as much as in the SRL setting group, yet it is clear from the interviews that students felt socially included in their classes. The lessons of the control group were characterized by less group work being done, as the use of group work in this setting depended much more on the instructional practices of the individual teachers.

“In some subjects we have group work. And then you don't decide for yourself who you're in the group with, but that they assign you at random.” (S_18_C, pos. 90)

However, it is also clear that, in certain situations, especially in the SRL setting group, there were issues in terms of social relatedness. For example, the SRL learning setting could also lead to students being disturbed by their peers when it became too noisy in the class or if students were not all working on the same content. Furthermore, not all students were interested in permanent collaboration or group work:

“Yes, actually they always ask me. I'm also happy to help, but sometimes it's a bit tedious. And the teachers are still there for something.” (S_2_T, pos. 152)

## 7 Discussion

The present study aimed to investigate the development of vocational students' motivational regulation over time and evaluate the effect of an SRL-promoting learning environment on their motivation. The study sought to extend previous research by considering situational and dispositional motivation to better understand the development of students' motivation over time. In addition, qualitative data were used to shed light on the different motivational states, in line with Deci and Ryan [39], and the factors promoting and impeding motivation from the students’ perspectives.

### 7.1 Students’ motivational development

Firstly, we analyzed the development of vocational students’ dispositional and situational motivation and whether the SRL-promoting setting had an impact on this development. Linear mixed-effect models revealed a significant decrease in identified regulation in the control group over the first year of vocational education. Additionally, weekly measures over the first 14 school weeks (one semester) showed a significant decrease in identified regulation in the control group. Therefore, both the fine- (weekly) and coarse-grained (annual) measurements produced the same results, suggesting that the situational decrease in identified regulation in the control group seems to become generalized over time.

The significant decrease in identified regulation over time in the control group is in line with previous research showing a decrease in autonomous motivation during secondary education [35,90]. The constant level of motivation in the SRL setting group might indicate an effect of the SRL setting on motivation. In addition, students in the SRL setting reported a significant increase in introjected regulation over the first 14 weeks of vocational education, but this effect disappeared in the second semester, leaving no significant effect in the annual measurement.

On the contrary, the annual measurements, showed a significant increase in amotivation in both groups, which was not apparent in the first semester. This result is in line with other results on amotivation suggesting that amotivation increases over time [58,91]. However, it remains unclear to what extent the satisfaction of students’ psychological needs prevents amotivation, because even students with the required competencies and personal control beliefs may still be amotivated due to a lack of energy or lack of perceived value [92].

Overall, in both groups, the small average change in motivation regulation during the first 14 weeks (situational development) and the first year of vocational education (dispositional development) is in line with previous research [5]. As suggested by Gillet, Vallerand [93], students’ motivation seems to slowly stabilize during adolescence. Furthermore, as suggested by the SEFT, contextual changes seem to lose their influence with age. Therefore, most of our hypotheses had to be rejected due to the absence of changes in motivation. This lack of change implies that future intervention approaches to promote academic motivation should aim to alter the generalized motivation patterns to a greater extent. For instance, the SRL-promoting learning environment as an indirect intervention implemented in this project could be complemented by the use of direct socio-psychological interventions [94]. However, as a difference between the two groups, it should be highlighted that the stability in the SRL setting group (i.e., the identified regulation remains stable) can be interpreted as a positive outcome of the SRL setting.

Secondly, as recently requested by Ryan and Deci [68], interview data were analyzed to better understand the factors motivating students in everyday life. The qualitative data showed that students in both groups reported similar motivational experiences. In terms of intrinsic regulation, the students mainly mentioned positive emotions and interest associated with learning activities. Regarding identified regulation, both groups highlighted the instrumental purpose of the learning activity (i.e., enabling active participation in society) as important.

However, introjected and external forms of regulation were also addressed in the interviews. Specifically, internal pressure (e.g., guilty conscience) and the associated negative emotions and self-perceptions emerged as relevant factors driving students' learning behavior. Additionally, the importance of formative and summative feedback from different actors (e.g., teachers, parents, apprenticeship companies) was highlighted in most interviews. Overall, from the qualitative data, it was evident that different aspects simultaneously motivate students’ learning behavior and that various forms of regulation coexist within students [45,95,96]. Although these different factors and regulation forms may affect learning activities to varying degrees (e.g., [1]), they collectively contribute to learning activities and should be taken into account when examining motivational experiences. Future research and practice should, thus, focus on the interaction of these different factors and regulation forms, as different combinations might attenuate or qualitatively alter the associations with academic outcomes [97].

Thirdly, both the quantitative and the qualitative data highlight the broad similarities between the two groups. Both groups showed little change in motivation over time, and their motivational experiences were similar in terms of their causes and effects (e.g., emotions). Therefore, for both research and practice, it is important to prevent a motivational decline in the early stages of education.

In addition, according to the qualitative data, the importance of external regulation was clearly emphasized by the students in both groups, although this was not evident to the same extent in the quantitative data (*M*_t1/t2_ = 3.40). Therefore, even in an SRL-promoting learning environment, external incentives are of great importance. Research on learning and instruction highlights the undermining phenomenon of external rewards (e.g., money; [98]). However, this effect has been challenged by researchers (e.g., [[95], [99]]) in the workplace, because payment in this context has a functional relevance that is interpreted individually and is crucial for the quality, quantity, and persistence of effort [100]. In this context, it is crucial to address the specific setting of vocational education, which is located in the transition between school and work. Therefore, further research on the functional significance of rewards for vocational students is needed.

Finally, the qualitative and quantitative data revealed that the four motivational forms of regulation can coexist within individuals [96]. In contrast, only a few moments of amotivation were described in the qualitative data. This result is in line with the quantitative data, where amotivation ranged between *M*_t0_ = 1.67 to *M*_t1_ = 2.01, meaning students answered “strongly disagree” to “rather disagree” for items on their levels of amotivation. This is an encouraging finding, as it suggests that there is an intention to learn among vocational students rather than a lack of intention.

However, a significant increase in amotivation was identified in both groups over the first year of vocational education. This result must be critically questioned due to the COVID-19 pandemic, as the increase was not yet visible in the first semester. As a result of the school closure during the second semester, students participated in distance learning for twelve weeks, impacting both groups equally. This situation may have had a negative impact on amotivation in the annual measurements. However, teachers in vocational education should be aware of possible increases in amotivation and try to motivate students by providing meaningful tasks. In future research, the coexistence of different motivation regulations within students should be investigated by using person-centered approaches (e.g., latent profile analysis), as a multifaceted view on motivation may provide insight into the causes and effects among different student groups [44,95].

### 7.2 Promoting and impeding factors

In addition to students' descriptions of their motivational experiences, we identified the promoting and impeding factors perceived by students in the qualitative data. Importantly, these factors contribute to a better understanding of students' motivational development. The three basic psychological needs emerged as central determinants of students’ perceptions of motivation in both groups—albeit to varying extents and with different explanations. Consistent with previous research, the satisfaction of basic psychological needs promotes motivation, whereas need frustration is regarded as an impeding factor by students [39,101,102].

In the SRL setting, autonomy was particularly emphasized. Students appreciated autonomy in the planning and implementation of their learning but also in setting their own goals (e.g., giving them a sense of choice). These results are in line with various research on autonomy support in school and learning (e.g., [[103], [104]]). Students in the control group reported autonomy regarding their extracurricular learning (e.g., homework), indicating that the two groups had a different understanding of autonomy in vocational school. Students in the SRL setting, who were familiar with regular instruction from compulsory school, perceived a strong difference in the setting, whereas students in the control group also perceived a difference due to the structural conditions of vocational education (2 school days and 3 days in the apprenticeship company vs. 5 days in school). Consequently, students’ perceptions and interpretations of their autonomy are crucial for understanding their motivational development.

As suggested by previous research, autonomy has various dimensions [105]. For students in high school, autonomy refers to following teachers' instructions and completing assignments, whereas in university, the dimensions of autonomy are more diverse [106]. This multidimensionality of autonomy may also be relevant in the present study, as the ability to plan extracurricular activities and independently complete a task in class was perceived as providing autonomy in the control group, whereas autonomy in the SRL setting was perceived in a more multifaceted manner (e.g., planning learning activities, breaks, etc.). This result indicates the possibility of distinguishing different facets within a single need (i.e., autonomy) to identify nuances (refinement of basic psychological needs; [39]). Overall, future research should both quantitatively and qualitatively examine and analyze students’ understanding of autonomy and its contribution to motivation, emphasizing the aspect of inter- and intraindividual differences. In terms of practice, these results imply that teachers should discuss the need for autonomy with their students and adapt their teaching accordingly.

Regarding relatedness, students in the SRL setting perceived having more opportunities to interact with peers as an advantage of the learning setting, and they valued the relationships with peers, teachers, and coaches. In addition, out-of-school actors also proved to be highly relevant for the students’ motivation to learn. In the control group, the fulfillment of the need for social relatedness was much more dependent on individual teachers and their instruction than in the SRL setting. Therefore, the qualitative data indicate that the SRL setting provides students with more options to fulfill their individual needs for relatedness.

No differences were expected between the groups in terms of relatedness to family and the apprenticeship company. The existing research does not show any differences in terms of the beneficial effects of different sources of relatedness on motivation [[107], [108], [109]], and all sources (peers, teachers, parents, training company) were considered important by students. Therefore, in terms of practice, teachers should focus on building good relationships with students and providing support services if students need further help. Interestingly, although the learning coaches were considered important by the students in the SRL setting, the motivational aspects of learning seemed to play a minor role in the coaching sessions. This result provides an indication that the promotion of motivation must be systematically anchored in the coaching sessions, and this concept should be verified by future research.

In both groups, the fit of the task difficulty to individuals’ competency was also highlighted a key motivating factor for learning. The satisfaction of the need for competence serves as a positive reinforcer for learning behavior, and this reinforcer can also have an impact across different subjects. Tasks that are too difficult may lead to negative emotions, thus producing avoidance behavior. In relation to these situations, students in both groups wanted more individualization. However, single events of a mismatch between task difficulty and competence (e.g., illustrated by a low grade) can also serve as motivators by encouraging students to learn from their mistakes and to want to do better next time. This result is consistent with previous findings highlighting the advantages and disadvantages of evaluative messages for different students [110].

Consequently, individual interpretation seems to be crucial in determining whether the misfit between task difficulty and competence is considered negative or positive, highlighting the importance of considering interindividual differences between students. A learning setting that is considered fulfilling for Student A in terms of their needs for autonomy, competence, and social relatedness may not be perceived as fulfilling for Student B in one or more of these dimensions. Further research should shed light on these interindividual differences. For example, a person-centered perspective may allow for deeper insight into within-person combinations of need satisfaction and their associated outcomes [111,112]. In addition, practitioners should consider individual interpretations and provide support to individual students who struggle with negative feedback.

Taken together, all three basic psychological needs (autonomy, competence, and relatedness) are considered as promoting and impeding factors for motivation, revealing their multifaceted nature. This nature is particularly clear in the case of autonomy for students in the SRL setting. The multifaceted nature might explain the lack of differences in students’ motivational development in the two groups, as the students in the SRL setting were much more exposed to the challenges associated with more autonomy than the control group. In addition to the fact that students experienced more autonomy in the SRL-promoting learning environment and, thus, had to manage this autonomy, the results also underscore the asymmetrical relationship between need satisfaction and frustration. The absence of need satisfaction does not necessarily coincide with the presence of need frustration, but the presence of need frustration implies the absence of need satisfaction [39]. Future research should focus on this relationship and investigate what students find need-frustrating, while teachers should address what students report as need-supportive.

### 7.3 Limitations

Despite the strengths of this study, including its longitudinal design, and supplementary qualitative interview data, some limitations must be acknowledged. Firstly, as mentioned, the COVID-19 pandemic and a related 12-week school closure affected our study during the second semester. This equally impacted both the SRL setting and control group and may have influenced the outcomes of the dispositional questionnaire administered at the end of the school year. In addition, we were unable to continue our weekly measurements during the school closure.

Secondly, the discrepancy in granularity between the yearly and weekly measurements resulted in time being segmented differently [113], potentially affecting the comparability of these measurements and those of other studies. In the present study, we combined fine- and coarse-grained measurements, with temporal units defined as weekly and yearly intervals. This choice of time units could substantially influence the interpretation of results, given that time can be viewed as an artificial construct [114].

Thirdly, the present study enriched the quantitative with qualitative data. However, both forms of data were self-reported and did not focus solely on motivation, as it was particularly evident in the interviews, when few explicit questions were asked about motivation. In addition, we noted that it was difficult for students to express their motivation in the interviews. Most students struggled to make precise statements about their motivation and often made rather vague statements that required follow-up questioning. Therefore, more targeted questions and objective data (e.g., classroom observation; [115]) could offer deeper insights into the motivational development of vocational students. Additionally, the quantitative measures had relatively low Cronbach's alpha values at the beginning of vocational education, perhaps due to the fact that the students had just started vocational school. Future studies should, thus, include more items and also related constructs (e.g., psychological needs or internalization) to validate the constructs and mechanisms following a transition to a completely new context and to determine which intervention elements lead to specific intervention effects.

In terms of the fourth limitation, participation in the SRL-setting was on a voluntary basis. Therefore, it cannot be ruled out that the self-selection of some students might have influenced the effectiveness of the intervention, even if students did not differ regarding their self-reported motivation before the intervention. In addition, completion of the questionnaires was on a voluntary basis, which resulted in a reduced sample size (especially in the weekly measurements), and a possible self-selection bias cannot be completely excluded. Consequently, the results need to be carefully reviewed and validated with a larger sample. In addition, the implemented SRL setting was a structural intervention that aimed to change the school environment to better meet students’ needs. This approach changes the daily school life of students and teachers and requires adaption of the curriculum, environment, and teaching practice. Accordingly, the implementation must be carefully planned and requires broad teacher and school support to be successful.

In terms of the fifth limitation, the extensive duration of the study and the frequency of weekly measurements contributed to a high number of missing values. While maximum likelihood estimations are suitable for addressing missing data [116], the possibility of bias cannot be completely excluded. Moreover, we used single-item measures for the weekly measurements, which may be less reliable than multi-item measures [78,81].

Finally, it must be noted that the intervention aimed to foster SRL and not explicitly motivation. Based on theoretical considerations, it can be assumed that an SRL-promoting setting supports students' self-determined motivation (see [42]), but a more focused intervention may have a stronger impact on motivation. It also remains unclear which aspects of the SRL setting specifically impacted students’ motivation regulation.

## 8 Conclusion

Overall, our study contributes to research on motivation, specifically in examining the impacts of an SRL setting in vocational education on two different time scales. Although this study revealed that motivation in vocational education tends to remain stable over time, the SRL-promoting learning environment had positive effects on students’ identified regulation, as reflected in both the weekly and yearly measures. Moreover, the quantitative, and qualitative data shed light on the coexistence of different motivation regulations within students, as well as individual interpretations regarding the satisfaction of basic psychological needs. These results highlight the dynamic nature of motivation and motivational factors, which should be further discussed in relation to SDT. The study also highlights the importance of a clear rationale for the measurement time scale in educational contexts, as some effects may disappear or not yet be evident if the wrong measurement time scale is chosen.

The study provides a basis for future research on the development and promotion of vocational students’ motivation. However, due to potential aptitude-treatment interactions, interventions may not yield uniform effectiveness for all students. Therefore, more research is required to understand systematic differences in the effectiveness of these interventions based on student characteristics.

## Data availability statement

Data will be made available on request.

## Ethics statement

• This study was reviewed and approved by the Ethics Committee of the Faculty of Human Science of the University of Bern, with the approval number: 2019-07-00001.
• All participants and/or their legal guardians provided informed consent to participate in the study.
• All participants and/or legal guardians provided informed consent for the publication of their anonymized data.

## CRediT authorship contribution statement

**Held Tanja:** Writing – review & editing, Writing – original draft, Formal analysis, Data curation, Conceptualization. **Mejeh Mathias:** Writing – review & editing, Writing – original draft, Project administration, Formal analysis, Data curation, Conceptualization.

## Declaration of competing interest

The authors declare that they have no known competing financial interests or personal relationships that could have appeared to influence the work reported in this paper.

## Footnotes

1 In Switzerland, after compulsory education, students attend either vocational education and training (VET) within a dual-track system or general education (baccalaureate school or upper-secondary specialized school). VET provides the skills and knowledge required to work in a specific occupation and leads to a federal VET certificate or diploma; 10). Approximately 60 % of students attend a VET program [117]. In VET, students spend 2 days in vocational schools in classes with students in the same apprenticeship sector and 3 days in their companies for practical experience.

2 The differences in the alpha values between the measurement points might be explained by the fact that the first measurement point was at the beginning of the school year right after the transition to vocational education. Therefore, some items (e.g., “I work and learn in vocational education because I want my teacher to think that I am a good student.“) showed a lower correlation with the other items of the scale at the first measurement point compared to the second measurement point [74].

3 “S” stands for “student,” the number corresponds to the interview number (1–19), and “T” and “C” indicate whether the student belonged to the treatment (SRL setting) or control group.
