# Supplementary material for: Students’ motivational trajectories in vocational education: Effects of a self-regulated learning environment
Source: Heliyon. 2024 Apr 10;10(8):e29526. doi: 10.1016/j.heliyon.2024.e29526 (PMC11046111; doi:10.1016/j.heliyon.2024.e29526)
Supplement: Multimedia component 1 [file mmc1.pdf]

Appendix A

Questionnaire

Note: The original items have been adapted for the German language area by Müller et al. (2007) and Thomas and Müller (2011). All items presented here were translated into English. All items were adapted from the school context to the vocational school context.

Intrinsic regulation (Müller et al., 2007)

Ich arbeite und lerne in der Berufsschule, weil es mir Spass macht.

I work and learn in vocational education because it’s fun.

Ich arbeite und lerne in der Berufsschule, weil ich neue Dinge lernen möchte.

I work and learn in vocational education because I want to learn new things.

Ich arbeite und lerne in der Berufsschule, weil ich es geniesse, mich mit den Themen auseinanderzusetzen.

I work and learn in vocational education because I enjoy learning about the topics.

Ich arbeite und lerne in der Berufsschule, weil ich gerne über diese Themen nachdenke.

I work and learn in vocational education because I like to think about these topics.

Ich arbeite und lerne in der Berufsschule, weil ich gerne Aufgaben löse.

I work and learn in vocational education because I like solving tasks.

Identified regulation (Müller et al., 2007)

Ich arbeite und lerne in der Berufsschule, weil ich die Sachen, die ich hier lerne, später gut für meinen Job gut gebrauchen kann.

I work and learn in vocational education because the things I learn here will be useful for my later job.

Ich arbeite und lerne in der Berufsschule, um später eine bestimmte Ausbildung machen zu können (z.B. Weiterbildung oder Studium).

I work and learn in vocational education to be able to do a specific education later (e.g., further education or university studies).

Ich arbeite und lerne in der Berufsschule, weil ich damit mehr Möglichkeiten bei der Arbeitsplatzwahl habe.

I work and learn in vocational education because it gives me more options when choosing a job.

Ich arbeite und lerne in der Berufsschule, weil ich mit dem Wissen später einen besseren Job bekommen kann.

I work and learn in vocational education because I can get a better job later with the knowledge.

Introjected regulation (Müller et al., 2007)

Ich arbeite und lerne in der Berufsschule, weil ich sonst ein schlechtes Gewissen hätte.

I work and learn in vocational education because otherwise I would have a guilty conscience.

Ich arbeite und lerne in der Berufsschule, weil ich möchte, dass meine Lehrerin oder mein Lehrer denkt, dass ich eine gute Schülerin bzw. ein guter Schüler bin.

I work and learn in vocational education because I want my teacher to think that I am a good student.

Ich arbeite und lerne in der Berufsschule, weil ich möchte, dass die anderen Mitschülerinnen und Mitschüler von mir denken, dass ich ziemlich gut bin.

I work and learn in vocational education because I want the other classmates to think of me as being pretty good.

Ich arbeite und lerne in der Berufsschule, weil ich mich vor mir selbst schämen würde, wenn ich es nicht tun würde.

I work and learn in vocational education because I would be ashamed of myself if I did not.

External regulation (Müller et al., 2007)

Ich arbeite und lerne in der Berufsschule, weil ich sonst Ärger in meinem Lehrbetrieb bekomme.

I work and learn in vocational education because otherwise I would get into trouble at my training company.

Ich arbeite und lerne in der Berufsschule, weil ich sonst von zu Hause Druck bekomme.

I work and learn in vocational education because otherwise I get pressure from home.

Ich arbeite und lerne in der Berufsschule, weil ich sonst schlechte Noten bekomme.

I work and learn in vocational education because otherwise I get bad grades.

Ich arbeite und lerne in der Berufsschule, weil ich es einfach lernen muss.

I work and learn in vocational education because I just have to learn it.

Ich arbeite und lerne in der Berufsschule, weil ich gute Noten bekommen will.

I work and learn in vocational education because I want to get good grades.

Ich arbeite und lerne in der Berufsschule, weil ich sonst Schwierigkeiten bekommen würde.

I work and learn in vocational education because otherwise I would get into trouble.

Amotivation (Thomas & Müller, 2011)

Wenn der Lehrer oder die Lehrerin es nicht bemerkt, beschäftige ich mich mit anderen Dingen.

If the teacher doesn't notice it, I occupy myself with other things.

Ich lerne oft gar nicht im Unterricht.

I often don't study in class at all.

Der Unterricht ist mir egal.

I don't care about the lessons.

Müller, F. H., Hanfstingl, B. & Andreitz, I. (2007). *Skalen zur motivationalen Regulation beim Lernen von Schülerinnen und Schülern: Adaptierte und ergänzte Version des Academic Self- Regulation Questionnaire (SRQ-A) nach Ryan & Connell* [Scales for motivational regulation in student learning: adapted and supplemented version of the Academic Self-Regulation Questionnaire (SRQ-A) according to Ryan & Connell]. Alpen-Adria-Universität.

Thomas, A. E. & Müller, F. H. (2011). *Skalen zur motivationalen Regulation beim Lernen von Schülerinnen und Schülern* [Scales of motivational regulation in student learning]. Alpen-Adria-Universität

.

Table S1

Excerpt of the Interview Guide for the SRL Setting Group

**Narrative stimulus**	**(Follow-up) questions**
During the apprenticeship you are at school for 2 days and have individual learning time. In addition, you work in your apprenticeship company and have to do a lot of work for school at home. Your learning jobs with the associated exams guide you during 4 weeks.<br>Can you explain to me how you proceed during these 4 weeks to achieve the goals of your weekly plans and learning jobs, including the exams?	What motivates you to work on the set goals, learning jobs, and to study for exams? Can you give an example?<br>What challenges you when studying or working on your assignments and how do you deal with it?
Learn coaching:<br>Once every 4 weeks, there is a coaching session with your coach. Describe a typical learning coaching conversation? What role does feedback play for you?	To what extent are you motivated to continue working through the coaching sessions?<br>Do you set individual learning goals during the coaching session? What does such a learning goal look like?<br>Describe how you work with the coach on your school challenges?
Evaluation “Treatment”<br>Imagine you would like to recommend the “Treatment” concept to a younger colleague. What are the arguments for and against choosing “Treatment”?	How would you rate the quality of the support provided by the teachers? Why?<br>What should definitely be maintained at “Treatment”? Why?<br>What changes in “Treatment” would you like to see in order to facilitate your learning and working processes at school?
Extracurricular activities:<br>School is only a small part of your everyday life. Your apprenticeship company, your hobbies, your family and your colleagues are sometimes more exciting, more important and more tempting than the work you do for school. How do these areas influence you, but also your learning?	What motivates you to learn and complete your assignments outside of school?<br>What does it mean for you to set priorities in your everyday life with regard to school?<br>How do you deal with it when everything other than school seems important to you?

Table S2

Excerpt of the Interview Guide for the Control Group

**Narrative stimulus**	**(Follow-up) questions**
During your apprenticeship, you are in school for 2 days and do a lot of work at home. Can you explain to me how and why you do your assignments and prepare for exams?	What motivates you to work on the set goals, assignments, and to study for exams? Can you give an example?<br>What challenges you when studying or working on your assignments and how do you deal with it?
Support:<br>Maybe you've had trouble with the subject matter or getting organized at vocational school. Explain how you deal with these and other school challenges and who you ask for support.	To what extent do teachers support you in (structuring and organizing) your learning?<br>From your perspective, how can students in a class also help and support each other?<br>Describe how the feedback you receive in class from the teacher affects you in the learning process.
School and classroom development:<br>At school and during your lessons you will be trained in different areas. Imagine that you are allowed to change something in the classroom. What would you like to change?	Describe how you envision productive instruction/learning.<br>How do you think your skills have developed since you started in vocational school?<br>What arguments do you have for not completing vocational school in the "treatment class"?<br>What aspects of the vocational school make your learning easier / more difficult?<br>What would you change about the instruction or the school structure so that you could learn more effectively?
Extracurricular activities:<br>School is only a small part of your everyday life. Your apprenticeship company, your hobbies, your family and your colleagues are sometimes more exciting, more important and more tempting than the work you do for school. How do these areas influence you, but also your learning?	What motivates you to learn and complete your assignments outside of school?<br>What does it mean for you to set priorities in your everyday life regarding school?<br>How do you deal with it when everything other than school seems important to you?

Table S3

Excerpt of the Coding Scheme Based on Self-Determination Theory (Deci & Ryan, 2002)

Category	Subcategory	Description	Reference example
Intrinsic regulation		Demonstrating a behavior because of the activity itself and an inherent satisfaction.	"The subject itself interests me, everything we have here at school."
Identified regulation		Behavior is considered important and fulfills an instrumental purpose (e.g., learning for good grades).	"The grade, if it is good."
Introjected regulation		Behavior is primarily based on feelings like shame, guilt, or pride.	"If I do not do the assignments, I have a guilty conscience."
External regulation		Behavior is performed based on reward or punishment.	"Depending on your grade, you will receive a salary increase. That's great motivation!"
Amotivation		Lack of intention to act.	"I have not learned."
Promoting factors	Fit of autonomy	One's actions, thoughts, and feelings are self-endorsed and authentic.	"That you can manage your time very autonomously and that it is all very independent."
	Fit of competence	One's skills and expertise can be used and expanded.	"Simply solve the tasks and then I realize, 'Ah I can do that! Yes, that's fun!' And then it goes on."
	Fit of social relatedness	One connects with others and feels important to others.	"She is great, my coach. I like her very much. She helps, she supports me, and she believes in me despite the low grades."
Impeding factors	Misfit of autonomy	One feels a sense of pressure (e.g., the feeling of being pushed in an undesirable direction).	"If they overrun the lesson and you only have in your head, 'I want to go now,' then you are already not really listening anymore."
	Misfit of competence	One feels ineffective or even a failure and helpless.	"Actually, it annoys me when I can't do the task afterwards."
	Misfit of social relatedness	One feels social alienation, exclusion, and loneliness.	"There are also some small groups that have formed. The class is just not a normal class and has no cohesion like in a normal class."
