# Supplementary material for: Students’ motivational trajectories in vocational education: Effects of a self-regulated learning environment
Source: Heliyon. 2024 Apr 10;10(8):e29526. doi: 10.1016/j.heliyon.2024.e29526 (PMC11046111; doi:10.1016/j.heliyon.2024.e29526)
Supplement: Multimedia component 2 [file mmc2.pdf]

Appendix B

Table S1

Model parameters and goodness of fit for linear changes in intrinsic regulation for both groups

Effect	Intrinsic regulation SRL setting group	$ICC_{student\ level}$ = .57	$ICC_{class\ level}$ = .00
	Model 1	**Model 2**	Model 3
Fixed effects			
Intercepts	3.21 (0.07)***	3.22 (0.09)***	3.22 (0.9)***
Time[b]		0.00 (0.11)	0.00 (0.11)
Random effects			
Intercept		0.54	0.67
Time[b]			0.60
Residual		0.48	0.23
Goodness of fit			
AIC	227.03	215.16	218.85
BIC	232.24	225.58	234.49
logLik[a]	-111.51	-103.58***	-103.43
Marginal R^2		0.00	0.00
Conditional R^2		0.56	0.90
Effect	Intrinsic regulation Control group	$ICC_{student\ level}$ = .68	$ICC_{class\ level}$ = .00
	Model 1	**Model 2**	Model 3
Fixed effects			
Intercepts	3.06 (0.07)***	3.09 (0.09)***	3.09 (0.09)***
Time[b]		-0.13 (0.09)	-0.13 (0.09)
Random effects			
Intercept		0.64	0.74
Time[b]			0.50
Residual		0.42	0.22
Goodness of fit			
AIC	293.56	276.58	280.58
BIC	299.25	287.96	297.64
logLik[a]	-144.78	-134.29***	-134.29
Marginal R^2		0.01	0.01
Conditional R^2		0.70	0.92

Note. Standard errors in parentheses. Model 1 = baseline model, Model 2 = random intercept model with time, Model 3 = random intercept random slope model; AIC = Akaike information criterion; BIC = Bayesian information criterion; logLik = log-likelihood; ICC = intraclass correlation coefficient. [a]nested model comparison. *$p<.05$; **$p<.01$; ***$p\leq.001$. [b](0 = start of vocational school [August], 1 = end of first year of vocational school [June]).

Table S2

Model parameters and goodness of fit for linear changes in identified regulation for both groups

Effect	Identified regulation SRL setting group	$ICC_{student\ level} = 0.59$	$ICC_{class\ level} = 0.04$
	Model 1	**Model 2**	Model 3
Fixed effects			
Intercepts	4.30 (0.06)***	4.31 (0.08)***	4.31 (0.07)***
Time[b]		-0.07 (0.09)	-0.07 (0.09)
Random effects			
Intercept		0.47	0.56
Time[b]			0.48
Residual		0.39	0.19
Goodness of fit			
AIC	187.58	176.82	180.37
BIC	192.79	187.24	196.00
logLik[a]	-91.79	-84.41***	-84.18
Marginal R^2		0.00	0.00
Conditional R^2		0.59	0.90
Effect	Identified regulation Control group	$ICC_{student\ level} = 0.20$	$ICC_{class\ level} = 0.00$
	Model 1	Model 2	**Model 3**
Fixed effects			
Intercepts	4.19 (0.07)***	4.34 (0.09)***	4.34 (0.08)***
Time[b]		-0.33 (0.13)*	-0.33 (0.13)*
Random effects			
Intercept		0.36	0.58
Time[b]			0.89
Residual		0.67	0.26
Goodness of fit			
AIC	299.55	296.14	291.91
BIC	305.23	307.49	308.93
logLik[a]	-147.78	-144.07*	-139.96*
Marginal R^2		0.04	0.04
Conditional R^2		0.26	0.90

Note. Standard errors in parentheses. Model 1 = baseline model, Model 2 = random intercept model with time, Model 3 = random intercept random slope model; AIC = Akaike information criterion; BIC = Bayesian information criterion; logLik = log-likelihood; ICC = intraclass correlation coefficient. [a]nested model comparison. *p<.05; **p<.01; ***p≤.001. [b](0 = start of vocational school [August], 1 = end of first year of vocational school [June]).

Table S3

Model parameters and goodness of fit for linear changes in identified regulation in the overall group

Effect	Identified regulation	$ICC_{student\ level}$ = 0.37	$ICC_{class\ level}$ = 0.01	
	Model 1	Model 2	Model 3	**Model 4**
Fixed effects				
Intercepts	4.24 (0.05)***	4.32 (0.06)***	4.32 (0.05)***	4.32 (0.08)***
Time[b]		-0.21 (0.08)*	-0.21 (0.08)*	-0.07 (0.13)
SRL setting				0.02 (0.11)
Time[b] x SRL setting				-0.24 (0.17)
Random effects				
Intercept		0.44	0.57	0.57
Time[b]			0.72	0.72
Residual		0.55	0.23	0.23
Goodness of fit				
AIC	491.39	480.34	474.00	475.79
BIC	498.23	494.02	494.52	503.16
logLik[a]	-243.69	-236.17**	-231.00**	-229.90
Marginal R^2		0.02	0.02	0.03
Conditional R^2		0.40	0.90	0.90

Note. Standard errors in parentheses. Model 1 = baseline model, Model 2 = random intercept model with time, Model 3 = random intercept random slope model, Model 4 = interaction model. Bold = selected model. AIC = Akaike information criterion; BIC = Bayesian information criterion; logLik = log-likelihood. [a]nested model comparison. *p<.05; **p<.01; ***p≤.001. [b](0 = start of vocational school [August], 1 = end of first year of vocational school [June]).

Table S4

Model parameters and goodness of fit for linear changes in introjected regulation for both groups

Effect	Introjected regulation SRL setting group	$ICC_{student\ level}$ = 0.36	$ICC_{class\ level}$ = 0.07
	Model 1	**Model 2**	Model 3
Fixed effects			
Intercepts	2.73 (0.09)***	2.61 (0.11)***	2.61 (0.10)***
Time[b]		0.31 (0.15)	0.30 (0.16)
Random effects			
Intercept		0.55	0.74
Time[b]			0.90
Residual		0.69	0.30
Goodness of fit			
AIC	268.66	263.83	264.70
BIC	273.90	274.29	280.39
logLik[a]	-132.33	-127.92*	-126.35
Marginal R^2		0.03	0.03
Conditional R^2		0.40	0.90
Effect	Introjected regulation Control group	$ICC_{student\ level}$ = 0.48	$ICC_{class\ level}$ = 0.06
	Model 1	**Model 2**	Model 3
Fixed effects			
Intercepts	2.66 (0.08)***	2.58 (0.11)***	2.58 (0.11)***
Time[b]		0.11 (0.13)	0.12 (0.13)
Random effects			
Intercept		0.64	0.89
Time[b]			0.82
Residual		0.65	0.30
Goodness of fit			
AIC	339.33	332.54	336.18
BIC	345.00	343.89	353.20
logLik[a]	-167.66	-162.27**	-162.09
Marginal R^2		0.00	0.00
Conditional R^2		0.49	0.89

Note. Standard errors in parentheses. Model 1 = baseline model, Model 2 = random intercept model with time, Model 3 = random intercept random slope model; AIC = Akaike information criterion; BIC = Bayesian information criterion; logLik = log-likelihood; ICC = intraclass correlation coefficient. [a]nested model comparison. *p<.05; **p<.01; ***p≤.001. [b](0 = start of vocational school [August], 1 = end of first year of vocational school [June]).

Table S5

Model parameters and goodness of fit for linear changes in external regulation for both groups

Effect	External regulation SRL setting group	$ICC_{student\ level}$ = 0.60	$ICC_{class\ level}$ = 0.00
	Model 1	**Model 2**	Model 3
Fixed effects			
Intercepts	3.36 (0.07)***	3.30 (0.09)***	3.30 (0.08)***
Time[b]		0.09 (0.10)	0.07 (0.11)
Random effects			
Intercept		0.54	0.59
Time[b]			0.55
Residual		0.44	0.21
Goodness of fit			
AIC	211.16	201.70	202.00
BIC	216.35	212.08	217.57
logLik[a]	-103.58	-96.85***	-95.00
Marginal R^2		0.00	0.00
Conditional R^2		0.60	0.92
Effect	External regulation Control group	$ICC_{student\ level}$ = 0.57	$ICC_{class\ level}$ = 0.00
	Model 1	Model 2	**Model 3**
Fixed effects			
Intercepts	3.45 (0.07)***	3.51 (0.08)***	3.52 (0.07)***
Time[b]		-0.15 (0.10)	-0.15 (0.10)
Random effects			
Intercept		0.54	0.58
Time[b]			0.61
Residual		0.48	0.22
Goodness of fit			
AIC	282.78	271.55	268.89
BIC	288.47	282.92	285.96
logLik[a]	-139.39	-131.77***	-128.45*
Marginal R^2		0.01	0.01
Conditional R^2		0.56	0.91

Note. Standard errors in parentheses. Model 1 = baseline model, Model 2 = random intercept model with time, Model 3 = random intercept random slope model; AIC = Akaike information criterion; BIC = Bayesian information criterion; logLik = log-likelihood; ICC = intraclass correlation coefficient. [a]nested model comparison. *p<.05; **p<.01; ***p≤.001. [b](0 = start of vocational school [August], 1 = end of first year of vocational school [June]).

Table S6

Model parameters and goodness of fit for linear changes in amotivation for both groups

Effect	Amotivation<br>SRL setting group	$ICC_{student\ level}$ = 0.14	$ICC_{class\ level}$ = 0.08
	Model 1	Model 2	**Model 3**
Fixed effects			
Intercepts	1.77 (0.07)***	1.64 (0.09)***	1.64 (0.07)***
Time[b]		0.35*	0.37 (0.16)*
Random effects			
Intercept		0.33	0.47
Time[b]			0.90
Residual		0.64	0.24
Goodness of fit			
AIC	230.07	227.03	211.91
BIC	235.30	237.49	227.60
logLik[a]	-113.04	-109.52*	-99.96***
Marginal R^2		0.05	0.05
Conditional R^2		0.25	0.91
Effect	Amotiovation<br>Control group	$ICC_{student\ level}$ = 0.36	$ICC_{class\ level}$ = 0.07
	Model 1	Model 2	**Model 3**
Fixed effects			
Intercepts	1.88 (0.07)***	1.71 (0.09)***	1.70 (0.08)***
Time[b]		0.39 (0.12)**	0.40 (0.12)**
Random effects			
Intercept		0.51	0.60
Time[b]			0.79
Residual		0.61	0.25
Goodness of fit			
AIC	314.18	303.04	297.88
BIC	319.87	314.42	314.95
logLik[a]	-155.09	-147.52***	-142.94*
Marginal R^2		0.06	0.06
Conditional R^2		0.45	0.91

Note. Standard errors in parentheses. Model 1 = baseline model, Model 2 = random intercept model with time, Model 3 = random intercept random slope model; AIC = Akaike information criterion; BIC = Bayesian information criterion; logLik = log-likelihood; ICC = intraclass correlation coefficient. [a]nested model comparison. *$p<.05$; **$p<.01$; ***$p\leq.001$. [b](0 = start of vocational school [August], 1 = end of first year of vocational school [June]).

Table S7

Model parameters and goodness of fit for linear changes in amotivation in the overall group

Effect	Amotivation	$ICC_{student\ level}$ = 0.25	$ICC_{class\ level}$ = 0.07	
	Model 1	Model 2	Model 3	**Model 4**
Fixed effects				
Intercepts	1.83 (0.05)***	1.67 (0.07)***	1.67 (0.05)***	1.64 (0.08)***
Time[b]		0.38 (0.09)***	0.39 (0.10)***	0.38 (0.15)**
SRL setting				0.06 (0.10)
Time[b] x SRL setting				0.02 (0.20)
Random effects				
Intercept		0.43	0.54	0.54
Time[b]			0.85	0.85
Residual		0.63	0.25	0.25
Goodness of fit				
AIC	542.53	524.47	501.96	505.52
BIC	549.38	538.19	522.53	532.95
logLik[a]	-269.26	-258.23***	-244.98***	-244.76
Marginal R^2		0.06	0.06	0.06
Conditional R^2		0.35	0.91	0.91

Note. Standard errors in parentheses. Model 1 = baseline model, Model 2 = random intercept model with time, Model 3 = random intercept random slope model, Model 4 = interaction model. Bold = selected model. AIC = Akaike information criterion; BIC = Bayesian information criterion; logLik = log-likelihood. [a]nested model comparison. *p<.05; **p<.01; ***p≤.001. [b](0 = start of vocational school [August], 1 = end of first year of vocational school [June]).
