# Supplementary material for: Students’ motivational trajectories in vocational education: Effects of a self-regulated learning environment
Source: Heliyon. 2024 Apr 10;10(8):e29526. doi: 10.1016/j.heliyon.2024.e29526 (PMC11046111; doi:10.1016/j.heliyon.2024.e29526)
Supplement: Multimedia component 3 [file mmc3.pdf]

Appendix C

Table S1

Model parameters and goodness of fit for linear changes in intrinsic regulation for both groups

Effect	Intrinsic regulation SRL setting group	$ICC_{student\ level} = 0.43$	$ICC_{class\ level} = 0.30$
	Model 1	**Model 2**	Model 3
Fixed effects			
Intercepts	3.15 (0.04)***	3.08 (0.09)***	3.08 (0.08)***
Time (weeks)		0.00 (0.01)	0.00 (0.01)
Random effects			
Intercept		0.43	0.39
Time (weeks)			0.02
Residual		0.51	0.50
Goodness of fit			
AIC	513.91	437.22	442.84
BIC	520.93	454.79	474.47
logLik[a]	-254.95	-213.61***	-212.42
Marginal R^2		0.00	0.00
Conditional R^2		0.42	0.44
Effect	Intrinsic regulation Control group	$ICC_{student\ level} = 0.52$	$ICC_{class\ level} = 0.03$
	Model 1	**Model 2**	Model 3
Fixed effects			
Intercepts	2.92 (0.05)	2.97 (0.11)***	2.97 (0.10)***
Time (weeks)		-0.01 (0.01)	-0.01 (0.01)
Random effects			
Intercept		0.60	0.52
Time (weeks)			0.02
Residual		0.58	0.57
Goodness of fit			
AIC	628.98	532.44	532.21
BIC	636.03	546.54	553.37
logLik[a]	-312.45	-262.22***	-260.11
Marginal R^2		0.00	0.00
Conditional R^2		0.52	0.52

Note. Standard errors in parentheses. Model 1 = baseline model, Model 2 = random intercept model with time, Model 3 = random intercept random slope model; Bold = selected model. AIC = Akaike information criterion; BIC = Bayesian information criterion; logLik = log-likelihood; ICC = intraclass correlation coefficient. [a]nested model comparison. *p<.05; **p<.01; ***p≤.001.

Table S2

Model parameters and goodness of fit for linear changes in identified regulation for both groups

Effect	Identified regulation SRL setting group	$ICC_{student\ level}$ = 0.42	$ICC_{class\ level}$ = 0.00
	Model 1	Model 2	**Model 3**
Fixed effects			
Intercepts	3.19 (0.04)***	3.12 (0.08)***	3.13 (0.07)***
Time (weeks)		0.01 (0.01)	0.01 (0.01)
Random effects			
Intercept		0.38	0.36
Time (weeks)			0.03
Residual		0.45	0.43
Goodness of fit			
AIC	460.23	383.59	378.81
BIC	467.27	397.67	399.94
logLik[a]	-228.11	-187.79***	-183.41*
Marginal R^2		0.01	0.01
Conditional R^2		0.41	0.48
Effect	Identified regulation Control group	$ICC_{student\ level}$ = 0.40	$ICC_{class\ level}$ = 0.03
	Model 1	**Model 2**	Model 3
Fixed effects			
Intercepts	3.02 (0.05)***	3.14(0.10)***	3.15 (0.09)***
Time (weeks)		-0.02 (0.01)*	-0.03 (0.01)*
Random effects			
Intercept		0.53	0.44
Time (weeks)			0.03
Residual		0.64	0.63
Goodness of fit			
AIC	624.25	561.47	561.87
BIC	631.30	575.57	583.02
logLik[a]	-310.13	-276.73***	-274.93
Marginal R^2		0.02	0.02
Conditional R^2		0.42	0.45

Note. Standard errors in parentheses. Model 1 = baseline model, Model 2 = random intercept model with time, Model 3 = random intercept random slope model; Bold = selected model. AIC = Akaike information criterion; BIC = Bayesian information criterion; logLik = log-likelihood; ICC = intraclass correlation coefficient. [a]nested model comparison. *p<.05; **p<.01; ***p≤.001.

Table S3

Model parameters and goodness of fit for linear changes in identified regulation in the overall group

Effect	Identified regulation Overall group	$ICC_{student\ level}$ = 0.42	$ICC_{class\ level}$ = 0.04	
	Model 1	Model 2	Model 3	**Model 4**
Fixed effects				
Intercepts	3.10 (0.03)***	3.13 (0.07)***	3.13 (0.06)***	3.10 (0.19)***
Time (weeks)		-0.01 (0.01)	-0.01 (0.01)	0.01 (0.01)
SRL setting				0.02 (0.12)
Time x SRL setting				-0.03(0.02)*
Random effects				
Intercept		0.47	0.41	0.41
Time (weeks)			0.04	0.03
Residual		0.56	0.54	0.54
Goodness of fit				
AIC	1113.47	974.47	965.93	964.50
BIC	1121.90	991.33	991.23	998.23
logLik[a]	-554.73	-483.23***	-476.96**	-474.25*
Marginal R^2		0.00	0.00	0.03
Conditional R^2		0.42	0.49	0.48

Note. Standard errors in parentheses. Model 1 = baseline model, Model 2 = random intercept model with time, Model 3 = random intercept random slope model, Model 4 = interaction model. Bold = selected model. AIC = Akaike information criterion; BIC = Bayesian information criterion; logLik = log-likelihood; ICC = intraclass correlation coefficient. [a]nested model comparison. *p<.05; **p<.01; ***p≤.001.

Table S4

Model parameters and goodness of fit for linear changes in introjected regulation for both groups

Effect	Introjected regulation SRL setting group	$ICC_{student\ level}$ = 0.48	$ICC_{class\ level}$ = 0.37
	Model 1	**Model 2**	Model 3
Fixed effects			
Intercepts	2.77 (0.06)***	2.60 (0.21)***	2.60 (0.22)***
Time (weeks)		0.03 (0.01)**	0.03 (0.01)*
Random effects			
Intercept		0.59	0.69
Time (weeks)			0.04
Residual		0.68	0.67
Goodness of fit			
AIC	690.82	595.65	600.70
BIC	697.85	613.23	632.36
logLik[a]	-343.41	-292.82***	-291.35
Marginal R^2		0.02	0.02
Conditional R^2		0.50	0.52
Effect	Introjected regulation Control group	$ICC_{student\ level}$ = 0.41	$ICC_{class\ level}$ = 0.01
	Model 1	Model 2	**Model 3**
Fixed effects			
Intercepts	2.79 (0.06)***	2.77 (0.11)***	2.77 (0.12)***
Time (weeks)		0.01 (0.01)	0.01 (0.01)
Random effects			
Intercept		0.60	0.68
Time (weeks)			0.06
Residual		0.73	0.68
Goodness of fit			
AIC	689.76	627.03	623.61
BIC	696.81	641.14	644.76
logLik[a]	-342.88	-309.52***	-305.80*
Marginal R^2		0.00	0.00
Conditional R^2		0.41	0.47

Note. Standard errors in parentheses. Model 1 = baseline model, Model 2 = random intercept model with time, Model 3 = random intercept random slope model; Bold = selected model. AIC = Akaike information criterion; BIC = Bayesian information criterion; logLik = log-likelihood; ICC = intraclass correlation coefficient. [a]nested model comparison. *p<.05; **p<.01; ***p≤.001.

Table S5

Model parameters and goodness of fit for linear changes in introjected regulation in the overall group

Effect	Introjected regulation Overall group	$ICC_{student\ level}$ = 0.44	$ICC_{class\ level}$ = 0.07	
	Model 1	Model 2	Model 3	**Model 4**
Fixed effects				
Intercepts	2.78 (0.04)***	2.66 (0.09)***	2.66 (0.09)***	2.29 (0.30)***
Time (weeks)		0.02 (0.01)**	0.02 (0.01)*	0.03 (0.01)*
SRL setting				0.24 (0.19)
Time x SRL setting				-0.02 (0.02)
Random effects				
Intercept		0.63	0.72	0.71
Time (weeks)			0.05	0.05
Residual		0.71	0.67	0.67
Goodness of fit				
AIC	1376.66	1218.27	1213.14	1215.29
BIC	1385.09	1235	1238.43	1249.01
logLik[a]	-686.33	-605.14***	-600.57*	-599.65
Marginal R^2		0.01	0.01	0.02
Conditional R^2		0.45	0.49	0.49

Note. Standard errors in parentheses. Model 1 = baseline model, Model 2 = random intercept model with time, Model 3 = random intercept random slope model, Model 4 = interaction model. Bold = selected model. AIC = Akaike information criterion; BIC = Bayesian information criterion; logLik = log-likelihood; ICC = intraclass correlation coefficient. [a]nested model comparison. *p<.05; **p<.01; ***p≤.001.

Table S6

Model parameters and goodness of fit for linear changes in external regulation for both groups

Effect	External regulation SRL setting group	$ICC_{student\ level}$ = 0.54	$ICC_{class\ level}$ = 0.32
	Model 1	Model 2	**Model 3**
Fixed effects			
Intercepts	2.42 (0.06)***	2.29 (0.15)***	2.30 (0.18)***
Time (weeks)		0.01 (0.01)	0.01 (0.01)
Random effects			
Intercept		0.69	0.56
Time (weeks)			0.03
Residual		0.66	0.65
Goodness of fit			
AIC	685.41	577.86	581.63
BIC	692.43	595.41	613.22
logLik[a]	-340.71	-283.93***	-279.34*
Marginal R^2		0.00	0.00
Conditional R^2		0.54	0.55
Effect	External regulation control group	$ICC_{student\ level}$ = 0.46	$ICC_{class\ level}$ = 0.01
	Model 1	**Model 2**	Model 3
Fixed effects			
Intercepts	2.41 (0.06)***	2.48 (0.12)***	2.47 (0.11)***
Time (weeks)		0.00 (0.01)	0.00 (0.01)
Random effects			
Intercept		0.64	0.60
Time (weeks)			0.04
Residual		0.70	0.68
Goodness of fit			
AIC	695.37	614.22	616.24
BIC	702.42	628.32	637.39
logLik[a]	-345.68	-303.11***	-302.12
Marginal R^2		0.00	0.00
Conditional R^2		0.46	0.49

Note. Standard errors in parentheses. Model 1 = baseline model, Model 2 = random intercept model with time, Model 3 = random intercept random slope model; Bold = selected model. AIC = Akaike information criterion; BIC = Bayesian information criterion; logLik = log-likelihood; ICC = intraclass correlation coefficient. [a]nested model comparison. *$p<.05$; **$p<.01$; ***$p\leq.001$.

Table S7

Model parameters and goodness of fit for linear changes in amotivation for both groups

Effect	Amotivation SRL setting group	$ICC_{student\ level}$ = 0.38	$ICC_{class\ level}$ = 0.10
	Model 1	Model 2	**Model 3**
Fixed effects			
Intercepts	1.85 (0.05)***	1.84 (0.11)***	1.88 (0.16)***
Time (weeks)		0.00 (0.01)	-0.01 (0.02)
Random effects			
Intercept		0.47	0.39
Time (weeks)			0.04
Residual		0.61	0.57
Goodness of fit			
AIC	596.87	521.95	514.63
BIC	603.89	539.50	546.22
logLik[a]	-296.43	255.98***	-248.32**
Marginal R^2		0.00	0.00
Conditional R^2		0.38	0.47
Effect	Amotivation Control group	$ICC_{student\ level}$ = 0.51	$ICC_{class\ level}$ =0.12
	Model 1	**Model 2**	Model 3
Fixed effects			
Intercepts	2.14 (0.06)***	2.01 (0.21)***	2.01 (0.21)***
Time (weeks)		0.01 (0.01)	0.01 (0.01)
Random effects			
Intercept		0.60	0.61
Time (weeks)			0.02
Residual		0.69	0.69
Goodness of fit			
AIC	705.18	613.79	621.61
BIC	712.24	631.42	653.33
logLik[a]	-350.59	-301.90***	-301.80
Marginal R^2		0.00	0.01
Conditional R^2		0.50	0.51

Note. Standard errors in parentheses. Model 1 = baseline model, Model 2 = random intercept model with time, Model 3 = random intercept random slope model; Bold = selected model. AIC = Akaike information criterion; BIC = Bayesian information criterion; logLik = log-likelihood; ICC = intraclass correlation coefficient. [a]nested model comparison. *p<.05; **p<.01; ***p≤.001.

Figure 4

Situational Development: Intrinsic Regulation

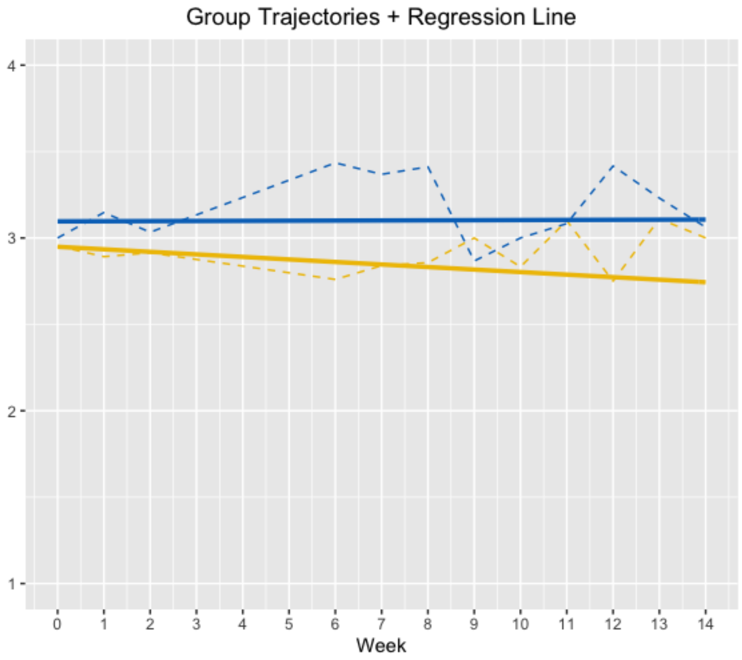

Note. Dashed line = aggregated values per measurement point; solid line = regression line; blue line = SRL setting group; yellow line = control group

Figure 5

Situational Development: External Regulation

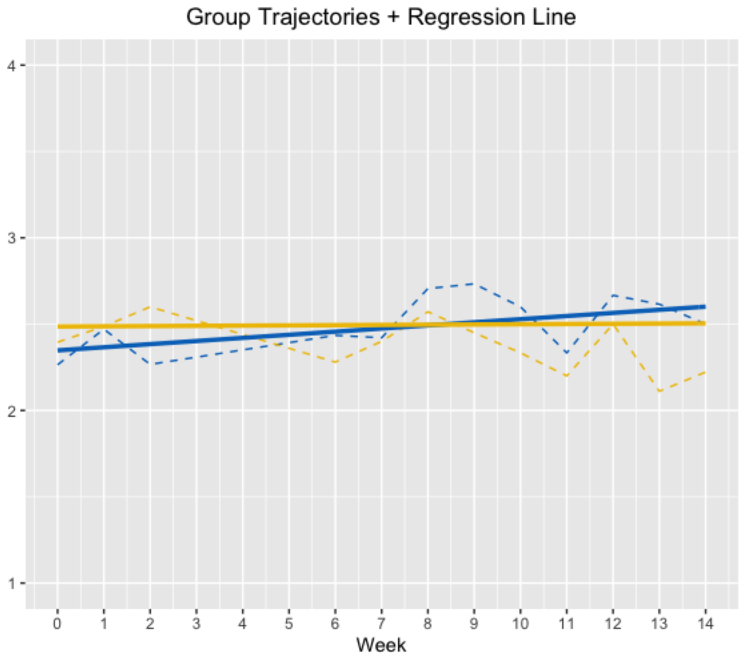

Note. Dashed line = aggregated values per measurement point; solid line = regression line; blue line = SRL setting group; yellow line = control group

Figure 6

Situational Development: Amotivation

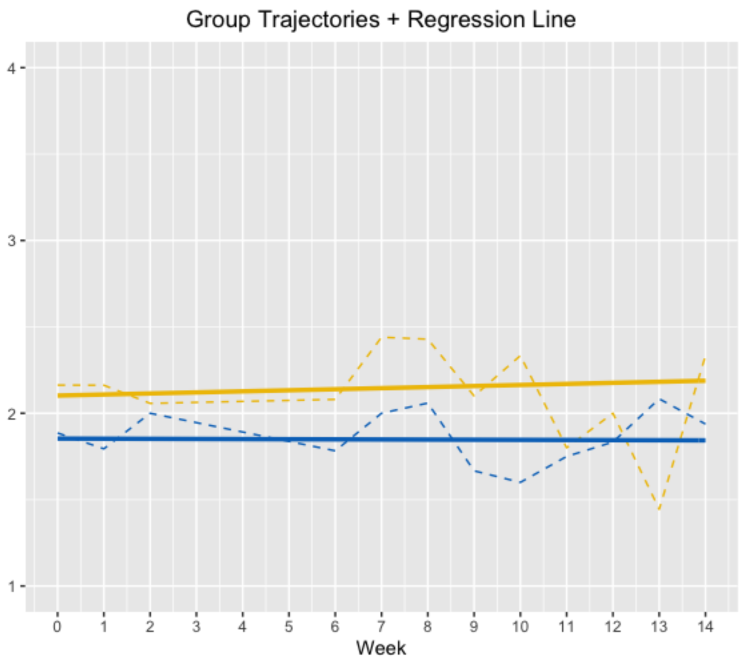

Note. Dashed line = aggregated values per measurement point; solid line = regression line; blue line = SRL setting group; yellow line = control group
